# Progresses and challenges of engineering thermophilic acetogenic cell factories

**DOI:** 10.3389/fmicb.2024.1476253

**Published:** 2024-08-30

**Authors:** Barbara Bourgade, M. Ahsanul Islam

**Affiliations:** ^1^Microbial Chemistry, Department of Chemistry-Ångström Laboratory, Uppsala University, Uppsala, Sweden; ^2^Department of Chemical Engineering, Loughborough University, Loughborough, United Kingdom

**Keywords:** acetogen, thermophile, Wood-Ljungdahl pathway, *Moorella*, *Thermoanaerobacter*, cell factory, genetic tools

## Abstract

Thermophilic acetogens are gaining recognition as potent microbial cell factories, leveraging their unique metabolic capabilities to drive the development of sustainable biotechnological processes. These microorganisms, thriving at elevated temperatures, exhibit robust carbon fixation abilities via the linear Wood-Ljungdahl pathway to efficiently convert C_1_ substrates, including syngas (CO, CO_2_ and H_2_) from industrial waste gasses, into acetate and biomass via the central metabolite acetyl-CoA. This review summarizes recent advancements in metabolic engineering and synthetic biology efforts that have expanded the range of products derived from thermophilic acetogens after briefly discussing their autotrophic metabolic diversity. These discussions highlight their potential in the sustainable bioproduction of industrially relevant compounds. We further review the remaining challenges for implementing efficient and complex strain engineering strategies in thermophilic acetogens, significantly limiting their use in an industrial context.

## 1 Introduction

As anthropocentric industrial activities accelerate the climate change, sustainable alternatives for manufacturing essential chemical commodities are urgently needed. Microbial biotechnology processes stand out as promising solutions due to their inherent robustness, adaptability, and less energy-intensive nature as compared to traditional chemical synthesis and fossil fuel-based methods (Ko et al., 2020; Cho et al., 2022). The development of reliable and efficient genetic tools, supporting various metabolic engineering strategies to expand and rewire microbial metabolic networks, has also allowed to further establish microbial cell factories as key production platforms. Acetogenic bacteria are becoming increasingly relevant in the current climate crisis context due to their autotrophic ability to utilize CO_2_ as their sole carbon source; thus, holding great promise to mitigate global warming by abating greenhouse gas emissions. In particular, these bacteria can assimilate a combination of H_2_, CO_2,_ and CO (i.e., syngas) (Liew et al., 2016) released by diverse industrial processes, hence offering the possibility to utilize industrial waste gas streams. Thus, acetogens can significantly contribute to industrial carbon capture and utilization efforts, alongside other non-biological strategies (McLaughlin et al., 2023; Yusuf and Ibrahim, 2023).

Although acetogens are very diverse in their metabolic capabilities, these anaerobic Gram-positive bacteria all rely on the Wood-Ljungdahl pathway (WLP) (Drake et al., 2008; Ragsdale, 2008), also known as the reductive acetyl-CoA pathway for carbon assimilation. They use the WLP to convert CO_2_ into the central metabolite acetyl-CoA, which is then channeled into both biomass and acetate formation. Operating at the thermodynamic limit of life (Schuchmann and Müller, 2014), acetogens have evolved intricate energy-conserving mechanisms to thrive autotrophically with the WLP. While significant progresses in terms of genetic engineering efforts and understanding of autotrophic processes have been achieved for mesophilic acetogens, their thermophilic counterparts remain largely understudied. However, thermophilic acetogens warrant a greater attention due to their unique advantages for large-scale cultivation and industrial bioprocesses, such as high turnover rates and reduced gas cooling requirements and contamination risks in bioreactors.

Despite their attractive characteristics, genetic and metabolic engineering of thermophilic acetogens for commodity bioproduction presents considerable challenges. Their metabolism is inherently constrained and the lack of efficient genetic tools complicates strain engineering. This review will discuss progresses and challenges of engineering thermophilic acetogens as microbial cell factories.

## 2 Thermophilic acetogenic isolates and their metabolism

### 2.1 Thermophilic acetogenic metabolism

**Figure 1:**
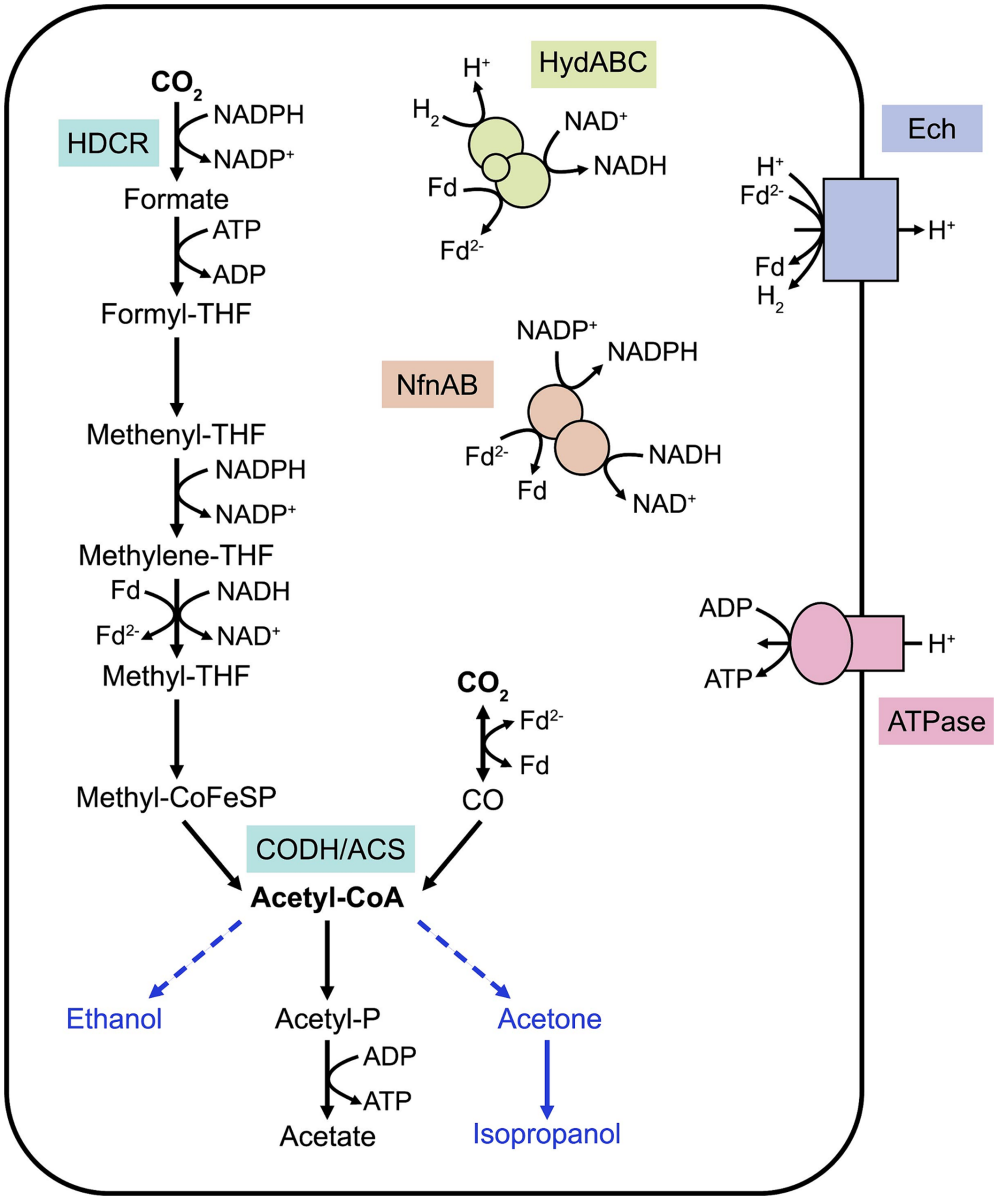
Simplified schematic of the Wood-Ljungdahl pathway in *M. thermoacetica*. The converging methyl and carbonyl branches allow CO_2_ conversion into acetyl-CoA, shuttled into biomass or acetate formation. Key enzymes of the WLP are represented. Known energy-conserving mechanisms are illustrated. Heterologous pathways for compound bioproduction are represented with blue arrows. Note that cofactor stoichiometry is not included in this figure and that cofactors and energy-conserving mechanisms differ between acetogens. HDCR, H_2_-dependent carbon dioxide reductase; CODH/ACS, CO dehydrogenase/acetyl-CoA synthase; THF, tetrahydrofolate; Fd, ferredoxin; HydABC, electron-bifurcating hydrogenase; NfnAB, electron-bifurcating transhydrogenase.

Acetogenesis can be defined as the ability to convert two molecules of CO_2_ into acetyl-CoA through the WLP (Schuchmann and Müller, 2016). Although the WLP is present in methanogens, this review focuses on homoacetogens which utilize this metabolic pathway for energy conservation. In these organisms, the WLP, described in details elsewhere (Ragsdale, 2008), consists of two converging branches, the methyl and carbonyl branches (Figure 1), which provide the methyl and carbonyl groups, respectively, for acetyl-CoA formation. This pathway requires an essential enzyme, the CO dehydrogenase/acetyl-CoA synthase (CODH-ACS) to form acetyl-CoA that is needed for biomass formation. Additionally, acetyl-CoA is converted into acetate, releasing one molecule of ATP. As formate conversion in the methyl branch requires one molecule of ATP, the net gain of ATP in the WLP is zero, placing acetogenic metabolism at the thermodynamic limit of life. Under autotrophy, the WLP is vital for carbon fixation, but this pathway is also active during heterotrophic growth as it participates into energy conservation and acts as a crucial electron sink.

Energy-conserving mechanisms have evolved to regenerate cofactors and are highly species-specific, as reviewed elsewhere (Basen and Müller, 2017). Briefly, most thermophilic acetogens rely on a membrane-bound energy-converting hydrogenase (Ech) to create a proton gradient across the membrane, which is utilized for ATP synthesis by an F_1_F_0_ ATPase (Figure 1). This proton translocation is also coupled to the oxidation of reduced ferredoxin, provided by the electron-bifurcating hydrogenase HydABC (Wang et al., 2013). NADPH, involved in the carbonyl branch of the WLP, is provided by the electron-bifurcating transhydrogenase NfnAB, with the concomitant conversion of reduced ferredoxin and NADH. Some of these processes and their stoichiometry remain unclear in thermophilic acetogens. In addition, acetogens can utilize a variety of electron carriers, enabling them to conserve energy by metabolizing diverse substrates such as pentoses, alcohols or organic acids (Basen and Müller, 2017). This metabolic flexibility is believed to be advantageous in their natural environments, where they compete with methanogens and sulfate-reducing bacteria. Acetogenic thermophiles important for industrial biotechnology applications are described in more details in the following sections.

### 2.2 *Moorella* sp.

To date, only a few acetogenic thermophiles have been isolated (Table 1). Most of these acetogens belong to the *Moorella* genus, with the first species, *M. thermoacetica*, isolated in the 1940s (Fontaine et al., 1942). *M. thermoacetica* has become the model *Moorella* species, and was key to describing and characterizing the WLP. Additional *Moorella* strains have since been isolated (Jia et al., 2023), and continuous discovery of new species highlights their diversity. Notably, the taxonomy of *Moorella* species still remains uncertain, as previously distinct species have recently been proposed to be the same species based on sequencing data (Redl et al., 2020).

**Table 1:** Thermophilic acetogens and their optimal growth conditions.

Species	Optimal temperature	Optimal pH	Notable features	References
**Bacteria**				
***Moorella* species**				
*M. caeni*	60–65°C	6.9	–	Santaella et al. (2023)
*M. glycerini*	58°C	6.3–6.5	–	Slobodkin et al. (1997)
*M. humiferrea*	65°C	7.0	Utilization of Fe(III) for electron shuttling	Nepomnyashchaya et al. (2012)
*M. mulderi*	65°C	7.0	–	Balk et al. (2003)
*M. perchloratireducens*	55–60°C	7.0	–	Balk et al. (2008)
*M. stamsii*	65°C	7.5	–	Alves et al. (2013)
*M. sulfitireducens*	60°C	6.5–7.0	Utilization of sulfite as electron acceptor	Slobodkina et al. (2022)
*M. thermoacetica*	55–60°C	6.9	Model *Moorella* species used to characterize WLP	Fontaine et al. (1942)
*M. thermoautotrophica*	56–60°C	5.7	Species not recognized by recent studies	Wiegel et al. (1981)
***Thermoanaerobacter* species**				
*T. kivui*	66°C	6.4	Narrow substrate range Fast doubling time under autotrophy	Leigh et al. (1981)
***Thermacetogenium* species**				
*T. phaeum*	58°C	6.8	Can revert the WLP in syntrophic cultures Reaches low ODs in anoxic autotrophic cultures	Hattori et al. (2000)
***Aceticella* species**				
*A. autotrophica*	46–50°C	6.0	Obligate autotroph	Frolov et al. (2023)
**Archaea**				
***Archaeoglobus* species**				
*A. fulgidus*	76–80°C	6.0	Described as a sulfate-reducing archaeon Adapted to grow on CO with the WLP	Stetter (1988), Henstra et al. (2007)

Selected notable features relevant for industrial applications are included.

*Moorella* sp. grow optimally at 55–60°C (Table 1) although pH and salinity parameters differ between species. They catabolize a variety of substrates, with several species reported to utilize methanol (Santaella et al., 2023). Both *M. thermoacetica* and *M. thermoautotrophica* are capable of microbial electrosynthesis (Yu et al., 2017; Chen et al., 2018; Ha et al., 2022), converting electricity and CO_2_ into high-value organic acids. This ability has, for example, been harnessed for acetate formation by *M. thermoautotrophica,* with the supply of electricity through metal electrodes, and further improved by embedding cells with carbon nanoparticules (Yu et al., 2017) and increasing cell permeability (Chen et al., 2018).

In addition to the energy-conserving mechanisms described above, the presence of quinones and cytochromes in *Moorella* sp. is unique among acetogens (Rosenbaum and Müller, 2021); however their roles remain unclear. Although a possible function as electron carriers in the electron transport chain, for example during lactate metabolism, has been proposed (Rosenbaum and Müller, 2021, 2023; Rosenbaum et al., 2021), further evidence is required to elucidate the role and importance of quinones and cytochromes for energy conservation in *Moorella* sp.

### 2.3 *Thermoanaerobacter* sp.

Among *Thermoanaerobacter* species, only *T. kivui* has been reported to fix CO_2_ through the WLP as other *Thermoanaerobacter* sp. do not contain CODH/ACS and hydrogen-dependent carbon dioxide reductase (HDCR) enzymes, essential to the WLP (Basen and Müller, 2017). Evolutionary emergence of acetogenic capabilities in this species remains unclear. In contrast to other thermophilic acetogens, *T. kivui* has a fast doubling time (~2 h) under H_2_ + CO_2_ conditions (Weghoff and Müller, 2016) and is naturally competent (Basen et al., 2018), simplifying laboratory cultivation and DNA uptake, which makes it particularly promising for industrial applications. It has been adapted for growth on CO and syngas (Weghoff and Müller, 2016), later shown to be supported by the presence of *cooS*, coding for a monofunctional CO dehydrogenase and essential for growth on CO (Jain et al., 2022). Recently, it has been reported that *T. kivui* can utilize mannitol in a CO_2_-dependent manner through expression of a mannitol-1-phosphate dehydrogenase (Moon et al., 2019, 2020).

While many unknowns remain regarding energy-conserving mechanisms and electron carriers involved in the WLP in *T. kivui*, genome analysis suggests that this organism relies on a proton (H^+^) gradient created by the Ech hydrogenase to drive ATP synthesis, similar to *Moorella* sp. (Hess et al., 2014). Electron carriers necessary for several enzymes involved in the WLP and energy conservation have been elucidated in cell-free extracts (Katsyv et al., 2021a), identifying, for example, NADP^+^-specificity of the methylene-THF dehydrogenase involved in the carbonyl branch of the WLP. However, the identity of electron carriers for other enzymes such as the electron-bifurcating hydrogenase HydABC remains unclear. In addition, the structure of the hydrogen-dependent carbon dioxide reductase (HDCR), which converts H_2_ and CO_2_ into formate in a high-turnover reaction, has now been elucidated with cryo-electron microscopy (Dietrich et al., 2022), providing a strong fundamental knowledge for this key enzyme. Interestingly, the formation of long HDCR filaments at the plasma membrane in *T. kivui* cells significantly enhanced enzymatic activity. Unsurprisingly, a Δ*hdcr* mutant was unable to grow autotrophically without formate supplementation in the medium (Jain et al., 2020). However, this phenotype was also observed under heterotrophy, highlighting the importance of the HDCR enzyme and the WLP for both autotrophy and heterotrophy.

### 2.4 *Thermacetogenium* sp.

Although *Thermacetogenium phaeum* is able to produce acetyl-CoA from CO_2_ with the WLP (Basen and Müller, 2017), this species exhibits poor growth in axenic cultures, reaching low maximal cell densities (Keller et al., 2019a). Instead, it preferentially grows in syntrophic cultures with the methanogen *Methanothermobacter thermautotrophicus*. In this syntrophic scenario, *T. phaeum* reverts the WLP for acetate consumption, a unique property not observed in other acetogens (Hattori et al., 2005). Genome analysis suggests that *T. phaeum*’s energy-conserving mechanisms and autotrophic metabolism differ significantly from other acetogens to accommodate for its bidirectional WLP (Oehler et al., 2012). In particular, ATP synthesis under both acetate formation and consumption raises several thermodynamic questions that are yet to be elucidated. A periplasmically oriented and quinone-dependent formate dehydrogenase has been proposed to allow WLP reversibility in *T. phaeum* (Keller et al., 2019a) although additional work is needed to elucidate energy-conserving mechanisms during both metabolic processes. Recently, the presence of pathways for methanol and ethanol degradation in *T. phaeum* have been proposed from proteomics and enzymatic activities, suggesting similar stoichiometries to the mesophilic acetogen *Acetobacterium woodii* (Keller et al., 2019b). This work also identified bacterial microcompartments involved in ethanolamine utilization in this thermophile. While progresses have been made toward understanding *T. phauem*’s metabolism, much more work is needed to uncover how this acetogen can revert the WLP, which has potential for industrial acetate valorization.

### 2.5 *Aceticella* sp.

The new thermophilic acetogenic species *Aceticella autotrophica* was recently isolated from a Russian terrestrial hot spring (Frolov et al., 2023). This species is the first obligate autotroph identified among acetogens and is unable to grow under heterotrophic conditions. Comparative genomics suggested that this strict autotrophic requirement results from the loss of genes involved in carbohydrate metabolism and sugar transport. Interestingly, while the species belongs to the *Thermoanaerobacterales* order, it has evolved unique features contrasting to its evolutionary counterparts. It shares the most similarity with *T. kivui* and contains a WLP gene cluster. Much more work is needed to characterize this new acetogen.

### 2.6 *Archaeoglobus* sp.

In addition to the aforementioned bacterial species, several mesophilic archaea (Loh et al., 2020; Schöne et al., 2022) are also acetogens. *Archaeoglobus fulgidus* is, to date, the only thermophilic archaeon reported to grow as an acetogen (Henstra et al., 2007). This species is primarily studied for its sulfate-reducing ability and piezophilic lifestyle (Oliver et al., 2020) but has been adapted to grow on CO (Henstra et al., 2007). CO adaptation eliminated *A. fulgidus*’s long lag-phase and was later investigated through transcriptomics analysis upon growth on CO (Hocking et al., 2014, 2015). This work proposed a scheme for energy conservation during the acetogenic growth of *A. fulgidus* by a F_420_H_2_:quinone oxidoreductase complex. Other *Archaeoglobus* species were explored but could not grow as acetogens, although key genes required for acetogenesis were present. Further work is thus needed to understand why *A. fulgidus* has the unique ability to grow as an acetogen and better-characterize its associated mechanisms.

## 3 Engineering thermophilic acetogens as microbial cell factories

### 3.1 Development and applications of genetic tools

Native acetogenic properties are extremely valuable for climate mitigation by fixing high CO_2_ concentrations in sustainable bioprocesses. However, to further expand their potential, reliable and efficient genetic tools must be developed to enable targeted strain engineering efforts that are crucial to rewire the metabolism for maximized compound bioproduction and heterologous pathway expression. Currently, genetic methods available for thermophilic acetogens are extremely limited. Successful genetic engineering has only been reported in *M. thermoacetica* and *T. kivui* although other acetogenic thermophiles have unique valuable properties.

In *M. thermoacetica*, genetic insertions have been performed using uracil/5-fluoroorotic acid (5-FOA) counterselection through deletion and reinsertion of *pyrF* (Iwasaki et al., 2013; Kita et al., 2013; Rahayu et al., 2017; Kato et al., 2021, 2024), encoding an orotodine 5′-phosphate decarboxylase. The Δ*pyrF* mutant becomes auxotrophic for uracil and resistant for 5-FOA, allowing transformant selection without antibiotic pressure. Reintroduction of *pyrF*, concurrently with the gene(s) of interest, restores uracil biosynthesis in the resulting mutant strain. This strategy has been applied to establish ethanol (Rahayu et al., 2017), acetone (Kato et al., 2021) and isopropanol (Kato et al., 2024) production in *M. thermoacetica* through expression of an aldehyde dehydrogenase, an acetone operon (consisting of a CoA transferase, a thiolase and an acetoacetate decarboxylase) and a secondary alchohol dehydrogenase, respectively. As these pathways branch from acetyl-CoA, disruption of acetate formation through deletion of the phosphotransacetylases PduL1 and PduL2 has been beneficial to redirect carbon flux toward compound biosynthesis (Kato et al., 2021). In addition to uracil auxotrophy, antibiotic selection by expressing a thermostable kanamycin resistant gene has also been reported (Iwasaki et al., 2013). This strategy has allowed the development of a self-replicating plasmid, harboring the pRKU1 replicon from *Thermotoga maritima* (Bourgade et al., 2022) and subsequently applied for ethanol production in proof-of-concept experiments. This self-replicating plasmid offers the possibility to rapidly and transiently test genetic constructs, ideal for, for example, CRISPR-Cas tools. Furthermore, to date, only one promoter, the strong constitutive promoter for glyceraldehyde-3-phosphate dehydrogenase (Kita et al., 2013) has been used for heterologous gene expression in *M. thermoacetica*. Thus, additional promoters are needed to expand the genetic toolbox to tailor target gene expression in this industrially important host.

In naturally competent *T. kivui*, a similar uracil/5-FOA counterselection technique has been adapted by deleting *pyrE*, encoding an orotate phosphoribosyltransferase involved in uracil biosynthesis (Basen et al., 2018). This method has primarily been used in fundamental studies to explore enzymatic functions of metabolic relevance. For example, Δ*hdcr*, Δ*cooS* and Δ*mtlD* mutants enabled to investigate formate formation, CO metabolism and mannitol consumption, respectively in *T. kivui* (Moon et al., 2019; Jain et al., 2020, 2022). *pyrE*-mediated genetic insertions have also allowed overexpression of the native *pfor1* (Katsyv et al., 2021b) and *mtlD* (Moon et al., 2019) genes, coding for a pyruvate:ferredoxin oxidoreductase and a mannitol-1-phosphate dehydrogenase, respectively, for protein purification from *T. kivui* cells. However, this method has not been reported for insertion and expression of heterologous genes yet but should allow successful pathway implementation in *T. kivui* in a similar manner to *M. thermoacetica*. A recent study successfully expressed the thermostable fluorescent reporter pFAST from a self-replicating plasmid, establishing a reporter assay for genetic part testing (Hocq et al., 2023). This tool was applied for promoter characterization to identify new strong constitutive promoters, such as the novel promoter pPta_Tkv_ for target gene expression in *T. kivui*. Interestingly, promoters from mesophilic acetogens were also functional in *T. kivui*, suggesting genetic part transferability. The authors isolated more stable versions of the replicon to promote plasmid propagation through adaptive laboratory evolution under antibiotic selection, significantly expanding *T. kivui* genetic toolbox.

Genetic tools are currently not available for other thermophilic acetogens but the development of such tools would greatly expand their industrial potential. It is worth noting that beyond microbial cell factories, thermophilic acetogens can also offer promising thermostable enzymes of industrial interest. For example, the recently characterized *T. kivui* HDCR (Dietrich et al., 2022) stands out as a promising biocatalyst for H_2_ storage. An alternative approach to using whole-cell biocatalysis has recently been reported to store H_2_ into formate (Schwarz and Müller, 2020). Additionally, the pyruvate:ferredoxin oxidoreductase can also be purified directly from *T. kivui* and is a promising auxiliary enzyme for enzymatic assays requiring reduced ferredoxin (Fd^2−^) difficult to provide otherwise (Katsyv et al., 2021b). Extensive research efforts on *A. fulgidus* have focused on characterizing various enzymes such as Argonaute (Manakova et al., 2024) or ferritin (Palombarini et al., 2021), highlighting its potential for enzymatic and therapeutic applications.

### 3.2 Engineering challenges

As described above, only two thermophilic acetogens have been genetically modified to date. While these efforts mark a significant step toward unlocking their potential, more complex genetic tools are needed to support extensive strain engineering of these thermophiles. Additionally, developing genetic methods for the other isolated thermophilic acetogens is crucial to accelerate their industrial potential for sustainable bioprocesses. However, several challenges remain for efficient genetic engineering of thermophilic acetogens. Successful transformation is currently hindered by multiple factors, such as difficulty of transformant selection and DNA entry into the host. Growth on plates has been reported as problematic for several acetogens (Sanford and Woolston, 2022). For instance, *M. thermoacetica* seems unable to grow on plates under antibiotic selective pressure (Bourgade et al., 2022). Instead, a rolling strategy in Hungate tubes has been used (Kita et al., 2013) which could result in high proportion of false transformants. In contrast, *T. kivui* is well adapted for growth on plates and can even tolerate brief exposure to oxygen at room temperature (Basen et al., 2018). The ability of other thermophilic acetogens to grow on plates is unknown but may restrict isolating positive transformants.

Many prokaryotes employ restriction-modification (RM) systems to protect themselves against invading foreign DNA (Vasu and Nagaraja, 2013). These systems recognize specific DNA sequences with associated methylation patterns to induce DNA cleavage; thereby, preventing foreign DNA entry into the host. Many acetogens possess these RM systems (Bourgade et al., 2021), which must be circumvented for a successful DNA entry into these hosts. For *M. thermoacetica*, bypassing of native RM systems has been reported by expressing three native genes encoding RM systems in an *E. coli* strain to protect cargo DNA prior to transformation into *M. thermoacetica* (Kita et al., 2013; Jia et al., 2023). This method may be applied for DNA insertion into other *Moorella* species or thermophilic acetogens.

Although genomic integration tends to enhance strain stability, it is often time-consuming and unsuitable for rapid construct testing or transient expression for CRISPR-based methods. Instead, self-replicating plasmids, able to propagate independently of chromosomal replication, are valuable genetic tools. However, these plasmids require compatible replicons for plasmid replication with the host’s machinery, often difficult to identify. Recently, a self-replicating shuttle vector was developed for *M. thermoacetica* using the pRKU1 replicon from *Thermotoga maritima* (Bourgade et al., 2022), which may be compatible with other closely related *Moorella* sp. However, additional work is needed to better understand this plasmid behavior in *M. thermoacetica*. *T. kivui* has previously been transformed with pMU131 replicon from *Thermoanaerobacterium saccharolyticum* (Basen et al., 2018). This replicon was later shown to be unstable at higher temperatures and subsequently improved through adapted laboratory evolution to increase its stability (Hocq et al., 2023).

In addition to replicons, genetic parts that are essential for achieving tuneable gene expression levels in thermophilic acetogens are poorly characterized. To date, only one promoter, from the glyceraldehyde-3-phosphate dehydrogenase has been used in *M. thermoacetica* to drive strong constitutive expression of heterologous genes (Kita et al., 2013). More promoters of varying strengths are needed to precisely control heterologous expression and metabolic output. Promoter characterization may prove difficult under thermophilic and anaerobic conditions, which render many fluorescent reporters non-functional. However, a reporter assay was recently developed for *T. kivui* with the O_2_-independent FAST system, allowing promoter characterization at high temperatures (Hocq et al., 2023) and is possibly compatible with other thermophilic acetogens. Ribosome-binding sites have yet to be characterized in these organisms but would be useful for achieving predictable translation levels.

Thermophily, while advantageous for metabolic efficiency and industrial applications, complicates genetic engineering work by limiting the pool of candidate enzymes that are functional at high temperatures. A thermostable *kanR* gene from *Streptococcus faecalis* has allowed kanamycin selection in *M. thermoacetica* (Iwasaki et al., 2013). Similarly, a thermostable acetone operon was engineered for *M. thermoacetica* by selecting candidate enzymes from other thermophilic prokaryotes (Kato et al., 2021). As exemplified by FAST (Hocq et al., 2023), exploring enzyme thermostability is crucial when working with thermophilic acetogens.

Finally, acetogenic metabolism is highly constrained by energy limitations and cofactor availability. These constraints significantly limit metabolic engineering possibilities, preventing expression of ATP-demanding pathways in these hosts. In particular, most pathways successfully implemented in acetogens stem from acetyl-CoA, therefore competing with ATP-yielding acetate formation. However, increasing target compound biosynthesis by abolishing acetate formation poses a problem for ATP synthesis during autotrophy and is often unviable for the host. For example, a *M. thermoacetica* Δ*pdul1Δpdul2::aldh* strain, producing ethanol instead of acetate, was unable to grow autotrophically on H_2_:CO_2_ (Takemura et al., 2021). Instead, ethanol was produced autotrophically with *Δpdul2::aldh* strain under CO supplementation, allowing ATP synthesis by decreased acetate formation using alternative routes. Another engineered *M. thermoacetica* strain required an additional electron acceptor to produce acetone autotrophically (Takemura et al., 2023). This work identified dimethyl sulfoxide as the strongest electron acceptor by enhancing ATP synthesis under H_2_:CO_2_ conditions.

## 4 Conclusion and outlook

Acetogens can fix CO_2_ into acetyl-CoA with the WLP, making them promising chassis organisms for large-scale biological CO_2_ fixation and compound bioproduction—a pivotal step toward mitigating climate change. Thermophilic acetogens offer additional advantages over their mesophilic counterparts by, for example, reducing gas cooling requirements and contamination risks in industrial bioprocesses. Several species with unique properties have now been isolated at temperatures above 55°C. However, most of them remain understudied, potentially due to the difficulty of cultivating and studying them under standard laboratory conditions. Consequently, significant knowledge gaps regarding their metabolism and physiology, in particular energy-conserving mechanisms remain. However, recent research efforts have started elucidating their metabolic processes, primarily in *M. thermoacetica* and *T. kivui*. Further work is, therefore, needed to fully understand their metabolism in order to design appropriate metabolic engineering strategies for industrial applications.

Moreover, *M. thermoacetica* and *T. kivui* have recently been engineered for heterologous compound biosynthesis and fundamental studies, respectively, paving the way for thermophilic acetogenic microbial cell factories. However, the genetic toolkit currently available for manipulating acetogens is limited, which further prevents complex strain engineering efforts. In particular, characterized genetic parts and thermostable enzymes are missing but are key elements for metabolic engineering. Significant genetic work is therefore needed to establish thermophilic acetogens as robust microbial cell factories for simultaneous CO_2_ fixation and compound biosynthesis.
